# Annual Banned‐Substance Review 18th Edition—Analytical Approaches in Human Sports Drug Testing 2024/2025

**DOI:** 10.1002/dta.70033

**Published:** 2026-02-17

**Authors:** Mario Thevis, Tiia Kuuranne, Hans Geyer

**Affiliations:** ^1^ Institute of Biochemistry, Center for Preventive Doping Research, German Sport University Cologne, am Sportpark Müngersdorf 6 Cologne/Bonn Germany; ^2^ European Monitoring Center for Emerging Doping Agents Cologne/Bonn Germany; ^3^ Swiss Laboratory for Doping Analyses, University Center of Legal Medicine, Genève and Lausanne, Centre Hospitalier Universitaire Vaudois and University of Lausanne Epalinges Switzerland

**Keywords:** doping, exposure, gene doping, LY305, sport

## Abstract

Alongside the considerable advances and accomplishments in drug research and development, the breadth of anti‐doping research topics has also continued to grow. This is particularly relevant not only to provide comprehensive information on new drug entities, drug metabolism and elimination, and/or the impact of administration and exposure routes on analytical test results but also to ensure the timely implementation of analytical methods that ensure the availability of relevant anti‐doping testing procedures and corresponding analytical data for routine doping control applications.

The 18th edition of the annual banned‐substance review on analytical approaches in human sports drug testing is dedicated to literature published between October 2024 and September 2025, and information published within these 12 months on established doping agents as well as new (potentially and evidently) relevant substances is reviewed and discussed, especially in the context of the World Anti‐Doping Agency's 2025 Prohibited List.

Topics of particular interest have been investigations into the metabolic fate and detection of anabolic agents, both anabolic–androgenic steroids and other anabolic substances such as selective androgen receptor modulators and protein‐based therapeutics negatively regulating the activin receptor signaling pathway, and detection strategies for numerous new drug candidates have been discussed and presented. Further, the trend toward expanding testing options with regard to gene doping practices including oligonucleotide‐based compounds (e.g., small interfering RNA, antisense oligonucleotides, etc.), transgenes, and gene editing practices continued also in 2024/2025, underlining the relevance for current and future sports drug testing programs.

## Graphical abstract

New insights into drug metabolism, distribution, elimination, and detection assays for drugs and methods of sports doping published between 2024 and 2025 are critically reviewed and evaluated in context with the Prohibited List 2025 as established by the World Anti‐Doping Agency.

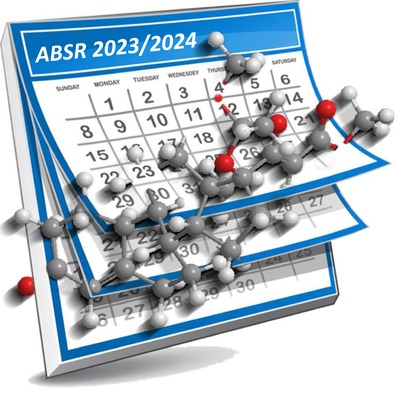

## 1 Introduction

Doping in sport is a multifaceted phenomenon and has been the subject of countless studies from numerous different scientific disciplines, highlighting attributes and characteristics of “doping” that are either to be considered as unique to sport or as principally observed in other professions and circumstances [1]. For example, investigations that aimed at identifying (and potentially managing) those factors that critically contribute to an athlete's tendency toward exploiting illicit means to enhance performance have corroborated not only the relevance of the athletes' vulnerability [2, 3], vulnerable or grandiose narcissism and self‐compassion [4], and the strive for perfectionism (rather than excellencism) [5], but also the importance of an implicit anti‐doping attitude that correlates with an athlete's diligence in avoiding unintentional doping scenarios [6]. In the context of performance optimization strategies, nutritional supplements are commonly considered means, with 50%–70% of Olympians and Paralympians resorting to the legitimate use of such products [7]. However, findings of products contaminated with prohibited substances that can lead to adverse analytical findings (AAFs) in sport are recurrently reported [8, 9]; an issue of growing concern for athletes that also applies to, e.g., meat exhibiting trace residues of growth factors [10] or Chinese traditional patent medicine containing naturally occurring and yet classified substances [11].

Also, situations of unintentional/unknown exposure to prohibited substances and corresponding analytical test results have been revisited in various reports, further stressing the importance of balancing between the needs for analytical sensitivity (and thereby retrospectivity) and the importance of selectivity in minimizing AAFs not associated with doping scenarios [12, 13, 14, 15, 16, 17, 18, 19, 20], as well as the validity and applicability of the anti‐doping rules in force.

Complemented and facilitated by strategic factors such as the consideration of including advanced performance data interpretation into anti‐doping testing programs [21] or sample long‐term storage and retesting programs [22], sports drug testing via analytical chemistry approaches continues to be a central aspect of global anti‐doping efforts and supports the enforcement of regulations established by international anti‐doping organizations. In order to appropriately meet the evolving needs of comprehensive and sensitive testing as well as the inclusion of emerging substances and methods of doping while respecting economic factors, inadvertent drug exposure scenarios, the burden on athletes caused by doping control sample collection sessions, etc., incessant anti‐doping research, and test method development (or refinement) are vital elements of that endeavor [23, 24]. Most testing approaches have continued to employ sensitive and specific multianalyte analytical approaches utilizing on chromatographic–mass spectrometric systems [25, 26, 27], which, to date, still outperform potential alternatives based on, e.g., biosensors in the area of routine doping control analyses [28]. A trend toward an increasing use of dried blood spots (DBSs) has, however, been observed, reflected also by a considerable number of reports on sample collection strategies [29], the relevance of preanalytical sample quality [30], and assays being developed and assessed for their fitness‐for‐purpose in sports drug testing programs, both for elite athletes subject to routine doping controls (vide infra) but also recreational athletes in the context of public health–related investigations [31].

Updates on the World Anti‐Doping Agency's (WADA's) Prohibited List are, among other factors, commonly initiated by new information resulting from medicinal, pharmaceutical, physiological, as well as dedicated anti‐doping research. In 2025, the Prohibited List maintained its established structure, which is composed of 11 classes of banned substances (S0–S9 plus P1) and three categories of prohibited methods (M1–M3) (Table 1) [32]. Modifications from the 2024 edition of the Prohibited List [33] included primarily the addition of further examples to specific sections, with, e.g., the ryanodine receptor‐1‐calstabin complex stabilizers S‐107 and ARM 210 as examples to S0, the anti‐estrogen elacestrant to S4.2, the peptide referred to as mitochondrial open reading frame of the 12S rRNA‐c (MOTS‐c) to S4.4.1, the selective insulin receptor modulators S519 and S597 to S4.4.2, the diuretic xipamide to S5, and midodrine, and tesofensine to S6.B.

**TABLE 1:** Overview of prohibited substances and methods of doping according to the World Anti‐Doping Agency (WADA) Prohibited List of 2025.

	Class	Subgroup		Examples	Prohibited	
					At all times	In‐competition only
**S0**	Nonapproved substances			BPC‐157, dinitrophenol, S‐107, S48168 (ARM210), reldesemtiv, tirasemtiv	x	
**S1**	Anabolic agents	1	Anabolic androgenic steroids		x	
				Androstenediol, 1‐androstenediol, clostebol, danazol, dehydroepiandrosterone, metandienone, methyltestosterone, methyltrienolone, nandrolone, stanozolol, testosterone, tetrahydrogestrinone, trestolone	x	
		2	Other anabolic agents	Clenbuterol, osilodrostat, ractopamine, selective androgen receptor modulators, zeranol, zilpaterol	x	
**S2**	Peptide hormones, growth factors, related substances, and mimetics	1.1	Erythropoietin‐receptor agonists	Darbepoietin (dEPO), erythropoietins (EPO), EPO based constructs (EPO‐Fc, methoxy polyethylene glycol‐epoetin β [CERA]), peginesatide, EPO‐mimetic agents and their constructs (CNTO‐530, peginesatide)	x	
		1.2	Hypoxia‐inducible factor activating agents	Cobalt, daprodustat, IOX2, molidustat, roxadustat, vadadustat, xenon	x	
		1.3	GATA inhibitors	K‐11706	x	
		1.4	TGF‐β signaling inhibitors	Luspatercept, sotatercept	x	
		1.5	Innate repair receptor agonists	Asialo EPO, carbamylated EPO	x	
		2.1	Testosterone‐stimulating peptides (in males)	Chorionic gonadotrophin, luteinizing hormone, gonadotrophin‐releasing hormone and its agonist analogs (e.g., buserelin, deslorelin, gonadorelin, leuprorelin), kisspeptin	x	
		2.2	Corticotrophins and their releasing factors	Tetracosactide‐hexaacetate (Synacthen), adrenocorticotrophic hormone, corticorelin	x	
		2.3	Growth hormone (GH), its analogs and fragments	Lonapegsomatropin, somapacitan, somatrogon AOD‐9604, hGH 176–191,	x	
		2.4	GH‐releasing factors	GHRH and its analogs (CJC‐1293, CJC‐1295, sermorelin, tesamorelin) GHS (ghrelin, anamorelin, ipamorelin, macimorelin, tabimorelin) GHRPs (alexamorelin, GHRP‐1, GHRP‐2, etc.)	x	
		3	Growth factors and growth factor modulators	Fibroblast growth factors, hepatocyte growth factor, insulin‐like growth factors (e.g., IGF‐I), mechano growth factors, platelet‐derived growth factor, thymosin‐β4 and its derivatives (TB‐500), vascular‐endothelial growth factor	x	
**S3**	β_2_ Agonists			Fenoterol, higenamine, reproterol, salbutamol, vilanterol	x	
**S4**	Hormone and metabolic modulators	1	Aromatase inhibitors	Anastrozole, letrozole, exemestane, formestane, testolactone	x	
		2	Anti‐estrogenic substances [anti‐estrogens and selective estrogen receptor modulators (SERMs)]	Bazedoxifene, raloxifene, tamoxifen, toremifene, clomiphene, cyclofenil, fulvestrant	x	
		3	Agents preventing activin receptor IIB activation	Domagrozumab, stamulumab, bimagrumab	x	
		4	Metabolic modulators	AICAR, GW1516, insulins and insulin‐mimetics, meldonium, SR9009, trimetazidine	x	
**S5**	Diuretics and masking agents		Masking agents	Probenecid, hydroxyethyl starch, desmopressin	x	
			Diuretics	Acetazolamide, bumetanide, chlortalidone, furosemide, triamterene	x	
**S6**	Stimulants		Nonspecified stimulants	Adrafinil, amfetamine, benfluorex, cocaine, modafinil		x
			Specified stimulants	Cathine, ephedrine, etamivan, methylephedrine, methylhexaneamine, octopamine, pseudoephedrine, sibutramine, strychnine, tesofensine, tuaminoheptane		x
**S7**	Narcotics			Buprenorphine, fentanyl, morphine, pentazocine, tramadol		x
**S8**	Cannabinoids			Hashish, marijuana, synthetic cannabinoids that mimic the effects of THC		x
**S9**	Glucocorticoids			Betamethasone, dexamethasone, prednisolone		x
**M1**	Manipulation of blood and blood components	1	Administration or reintroduction of any quantity of blood	Autologous, homologous and heterologous blood, red blood cell products	x	
		2	Artificially enhancing the uptake, transport or delivery of oxygen	Perfluorocarbons, efaproxiral, hemoglobin‐based blood substitutes	x	
		3	Intravascular manipulation of blood or blood components by physical or chemical means		x	
**M2**	Chemical and physical manipulation	1	Tampering	Sample substitution, proteases	x	
		2	Intravenous infusion/injection	More than 100 mL per 12‐h period	x	
**M3**	Gene and cell doping	1	The use of nucleic acids or nucleic acid analogues that may alter genome sequences and/or alter gene expression by any mechanism. This includes but is not limited to gene editing, gene silencing and gene transfer technologies	DNA, RNA, siRNA	x	
		2	Use of normal or genetically modified cells			
**P1**	β‐Blockers			Acebutolol, atenolol, bisopropol, metoprolol	x^a^	x^a^

^a^ Depending on the rules of the international sport federations.

The collection of data to assess patterns of use of selected substances continued under the umbrella of the 2025 Monitoring Program [34]. Here, the recording of the prevalence of ecdysterone in urine (at all times) continued also in 2025, seconded by in vitro [35] investigations that demonstrated significant stimuli for hypertrophy of C2C12 myotubes and increased insulin‐like growth factor I (IGF‐I) mRNA expression by ecdysterone, as well as its in silico determined binding capacities to the estrogen receptor β and resulting (presumed) anabolic effects [36].

Further, the gonadotrophin‐releasing hormone analogs (in females under 18 years), hypoxen, and semaglutide monitoring continued also in 2025, both in‐ and out‐of‐competition to furnish the aimed dataset. Concerning hypoxen, advanced insights into the substance's pharmacology and thus potential for its impact on athletic performance [37], as well as additional information on the drug's composition [38], were provided, and the question whether or not other drugs with “anti‐hypoxic” properties might warrant consideration in monitoring programs was raised [39]. With regard to semaglutide (and GLP‐1 receptor agonists in general), the aspect of reduced lean body mass and the option to address this with myostatin–activin pathway inhibitors and/or anabolic agents (e.g., selective androgen receptor modulators [SARMs]) reinforce the potential relevance of drugs supporting optimized weight management among athletes [40, 41].

The 2025 Monitoring Program continued to include the recording of in‐competition use of the stimulants bupropion, caffeine, nicotine, phenylephrine, phenylpropanolamine, pipradrol, and synephrine and the narcotics codeine, dermorphin and its analogs, dihydrocodeine, hydrocodone, and tapentadol. In addition, the out‐of‐competition use of the narcotics fentanyl and tramadol (the classification of tramadol as prohibited in‐competition came into effect in January 2024) was introduced into the Monitoring Program. Conversely, while remaining a controversial issue in elite sport, thyroid hormones such as triiodothyronine and thyroxine or metabolites/biomarkers indicative of their use were not included in the 2025 Monitoring Program. A trend toward a decline in (self‐declared) use of thyroid hormones among Olympic athletes was reported, potentially reflecting the position that this class of drugs is not an effective ergogenic aid [42]. Whether or not β‐hydroxy β‐methyl‐butyric acid should be considered in anti‐doping was discussed by Camuto et al., who established an analytical assay allowing for determining commonly observed endogenously produced levels of the substance as well as post‐administration drug concentrations in urine [43]. With reported effects on mitochondrial biogenesis, post‐exercise recovery, fat vs. muscle mass ratio, etc., it was suggested to include the substance into future considerations of anti‐doping research.

In this continuation of the 17th edition of the Annual Banned‐Substance Review [23], literature published between October 2024 and September 2025 was evaluated and contextualized (Table 2), outlining the most recent thematic foci in anti‐doping research. Key topics centered around advancements in sports drug testing approaches, optimized selection of target analytes for prohibited compounds and/or confounding factors, as well as novel solutions based on new analytical instrumentations, plus growing interest in and consideration of alternative sample collection options, sample matrices, preparation, and analysis protocols.

**TABLE 2:** References to new data and/or improved screening and confirmation methods regarding human sports drug testing published in 2024/2025.

	Class	Subgroup		References			
				GC/MS (/MS)	LC/MS (/MS)	GC/C/or LC/IRMS	Complementary methods and general
**S0**	Nonapproved substances						[44]
**S1**	Anabolic agents	1	Anabolic androgenic steroids	67–70, 78	71, 74, 79, 83–85	80, 81	[45, 46, 47, 48, 49, 50, 51, 52, 53, 54, 55, 56, 57, 58, 59, 60, 61, 62, 63, 64, 65, 66, 67, 68, 69, 70, 71]
		2	Other anabolic agents	91	88, 90, 91		[72, 73, 74, 75, 76]
**S2**	Peptide hormones, growth factors, related substances, and mimetics	1.1	Erythropoietin‐receptor agonists	107, 109			[77, 78, 79, 80, 81, 82, 83, 84, 85]
		1.2	Hypoxia‐inducible factor activating agents				[86]
		2.1	Testosterone‐stimulating peptides		113		
		2.3	Growth hormone (GH), its analogs and fragments		115, 117, 118		[87, 88]
		2.4	GH‐releasing factors		114		
**S3**	β_2_ Agonists						[89, 90, 91, 92, 93]
**S4**	Hormone and metabolic modulators	3	Agents preventing activin receptor IIB activation		129, 130		[94]
		4	Metabolic modulators		134		[95, 96]
**S5**	Diuretics and masking agents						[91]
**S6**	Stimulants	A	Nonspecified stimulants		136, 137		[97]
		B	Specified stimulants		136, 137		[97, 98, 99, 100, 101]
**S9**	Glucocorticoids				142		[102]
**M1**	Manipulation of blood and blood components	1	Administration or reintroduction of any quantity of blood or blood products				[103, 104, 105, 106]
		2	Artificially enhancing the uptake, transport or delivery of oxygen		153		
**M2**	Chemical and physical manipulation	1	Tampering				[107]
**M3**	Gene and cell doping	1	The use of nucleic acids or nucleic acid analogues that may alter genome sequences and/or alter gene expression				[108, 109, 110, 111, 112]

## 2 Nonapproved Substances

This category of the WADA Prohibited List, referred to as “S0” [32], was created in 2011 and allows for pursuing findings of those substances that fulfill WADA's criteria justifying a prohibition in sports, but which are not covered by any other section of the document. More specifically, no current approval exists for these compounds for human therapeutic use by any governmental regulatory health authority, but availability, (pre)clinical data, and/or reported investigational use of some S0‐classified candidates such as BPC‐157 [44, 113, 114], ryanodine receptor‐1‐calstabin complex stabilizers (e.g., S‐107) [115], or troponin activators indicate the importance of appropriately updated test methods in routine doping controls. With regard to the troponin activators reldesemtiv and tirasemtiv, new information on biotransformation products was provided, obtained from administration studies with rats receiving a single transgastric dose of 10 mg/kg body weight of either substance [116]. Although administered species and delivery routes might limit the immediate transfer of the findings to human sports drug testing applications, the employed analytical approaches for blood and urine demonstrated fitness‐for‐purpose and data complementing those of previous human in vivo studies [117]. Following acetonitrile‐supported precipitation, blood and urine samples were subjected to liquid chromatography (LC)–electrospray ionization (ESI)–high‐resolution/accurate mass (HRAM) analysis. Here, target compounds were separated on a C‐18 analytical column (2.1 × 100 mm, 1.9‐μm particle size) using aqueous formic acid (0.1%) and acetonitrile as solvents A and B, respectively. Product ion mass spectra of relevant analytes were recorded in data‐dependent MS/MS mode on a quadrupole (Q)/orbitrap‐based analyzer, and structures were tentatively attributed, mostly postulating oxidation and glucuronidation for tirasemtiv, and the conversion of the amide residue of reldesemtiv into the corresponding carboxylic acid, defluorination, and/or conjugation. Within the monitored period of 72 h, urine provided superior detection options compared to blood.

## 3 Anabolic Agents

Anabolic–androgenic steroid (AAS) and SARM use among elite as well as recreational athletes has created a multitude of critical missions, challenges, and responsibilities spanning from (public) health concerns to legal and eventually also sports integrity aspects [45, 46, 47, 48, 49, 50, 51, 52, 53, 54, 55, 56, 57, 58, 59] At the same time, the issue of inadvertent exposure and corresponding AAFs appears to (still) apply predominantly to anabolic agents [15, 16, 17, 118], indicating the persisting relevance of both comprehensive doping controls and further optimization or refinement of testing approaches allowing for best‐possible result interpretation and management.

### 3.1 Anabolic–Androgenic Steroids

The trend of previous years with regard to anti‐doping research concerning AAS continued also in 2024/2025. Published studies primarily focused on an improved understanding of the prohibited drugs' metabolism, the optimized exploitation of monitoring biomarkers in a longitudinal manner, the use of complementary test matrices (e.g., capillary blood), and new or advanced means that facilitate the differentiation of natural and endogenously produced steroids from their synthetic analogs. Here, the number of chromatographic–mass spectrometric approaches outbalance by far complementary approaches based, e.g., on bioassay platforms, but those have also been revisited considering their nontargeted screening capabilities and thereby added value in supporting subsequent target‐testing or retesting programs conducted by anti‐doping organizations [119].

#### 3.1.1 Initial Testing Procedures and Studies on Metabolism

The plethora of AAS and, in addition, the impact of drug administration routes and regimens has necessitated continued research into the sensitive and robust analysis of known target analytes but also in‐depth investigations into drug metabolism patterns in order to provide state‐of‐the‐art detection methods in routine doping controls.

Steff et al., for example, revisited the biotransformation of 10 mg of orally administered metandienone in men [60]. Urine samples were collected before and up to 60 days post‐administration of metandienone and subjected to a comprehensive analysis targeting established as well as predicted metabolite structures, for which an extensive number of synthetic comparators was prepared. Urine samples were enzymatically hydrolyzed with β‐glucuronidase before undergoing liquid–liquid extraction (LLE), and metabolite identification and elimination profile analyses were conducted on GC‐quadrupole/time‐of‐flight (GC‐Q/TOF) and GC‐triple quadrupole (GC‐QqQ) instruments, respectively. Those instruments comprised either a fused silica capillary column of 30‐m length (5% phenyl‐methylpolysiloxane, 0.25‐mm inner diameter, 0.25‐μm film thickness) and low energy electron ionization (EI; GC‐Q/TOF) or a setup consisting of a 17‐m dimethyl polysiloxane capillary column (0.20‐mm inner diameter, 0.11‐μm film thickness) and 70‐eV EI on GC‐QqQ. The obtained data allowed for confirming previously suggested and tentatively identified metabolic products such as 17,17‐dimethyl‐18‐nor‐5β‐androst‐13‐en‐3α‐ol and, more importantly, provided evidence for the excretion of 17α‐hydroxymethyl‐17β‐methyl‐18‐nor‐5β‐androsta‐1,13‐dien‐3α‐ol, whose detection window indicated considerable retrospectivity for sports drug testing purposes.

Comparing both in vitro [61] and in vivo [62] metabolism, Zheng et al. focused on investigating the AAS oxymetholone and methasterone using a GC‐Q/orbitrap‐based analyzer. For the in vitro metabolism study [61], the drugs were subjected to human liver S9 fraction and relevant co‐substrates that allow for phase I and II biotransformation reactions. The obtained products were separated into unconjugated metabolites, glucuronides, and sulfoconjugated analytes, where the phase II metabolic products were either enzymatically hydrolyzed or chemically cleaved, respectively, prior to trimethylsilylation and GC‐Q/orbitrap analysis. The analytical instrument featured a 25‐m dimethyl polysiloxane capillary column (0.20‐mm inner diameter, 0.11‐μm film thickness) interfaced via EI (70 eV) to the mass spectrometer, which was operated in full scan mode. The in vivo experiments involved two male volunteers each, who administered a single oral dose of either 50 mg of oxymetholone or 10 mg of methasterone, and urine samples were collected prior to and up to 50 days post‐administration. The sample preparation and analysis protocol were identical in both studies. For oxymetholone, six urinary metabolites were reported, three of which were also observed following the in vitro incubation experiment, and the glucuronic acid–conjugated species proved to offer detection windows of up to 4 days, with a new and tentatively identified structure exhibiting a 16‐hydroxylation (2‐methylene‐17α‐methylandrostane‐16ξ,17β‐diol‐3‐one) being potentially of particular interest for routine doping controls. Concerning methasterone, 16‐hydroxylation was also observed, both in vitro and in vivo, and more importantly, the considerable retrospectivity offered by targeting 18‐nor‐17β‐hydroxymethyl‐2α,17α‐dimethyl‐5α‐androst‐13‐en‐3‐one was shown with detection windows > 50 days using routine doping control methods.

In an investigation by Montes de Oca Porto et al., the in vivo metabolism of the AAS 17α‐methyl‐19‐nortestosterone was revisited [63]. Urine samples collected after the oral administration of a single dose of 10 mg of 17α‐methyl‐19‐nortestosterone were subjected to β‐glucuronidase–based hydrolysis, LLE, and trimethylsilylation, followed by GC‐EI‐MS analysis. Gas chromatography (GC) was done using a 17‐m dimethyl polysiloxane capillary column (0.20‐mm inner diameter, 0.11‐μm film thickness), interfaced via EI at 70 eV to a single quadrupole MS. The study corroborated the relevance of established A‐ring–reduced metabolites for doping control purposes, but also presented data suggesting the formation of a carbon‐18‐hydroxylated species (17α‐methylandrostane‐17β,18‐diol‐3‐one), which could complement analytical assays for AAS in anti‐doping laboratories.

With methyltrienolone, another 17α‐methyl‐19‐norsteroidal compound was studied with regard to its in vitro biotransformation by Yan et al. [64] using human liver microsomal preparations. Seven phase I and eight glucuronides were detected, featuring primarily metabolic reactions including 17‐epimerization, reduction, hydroxylation, and combinations thereof, offering a variety of new potential target analytes. Structural assignments were accomplished using LC–MS–based approaches, employing a C‐18 analytical column (2.1 × 200 mm, 1.9‐μm particle size) operated with 10 mM ammonium formate (solvent A, containing 0.05% formic acid) and methanol (solvent B, containing 0.1% formic acid) for chromatographic separation of the methyltrienolone metabolites. Via ESI in positive mode, the hydroxylamine‐ or methoxylamine‐derivatized analytes were measured using a Q/orbitrap mass analyzer in parallel reaction monitoring (PRM) mode, and obtained product ion mass spectra suggested, among others, the formation of the 17‐hydroxymethyl‐18‐nor‐analog of methyltrienolone that, in accordance with other 17‐methylated AAS, could represent an important long‐term metabolite for routine doping controls. To which extent this metabolite is formed in vivo is yet unclear, but its consideration in ITP might be advisable.

Similar to methyltrienolone, LC–ESI–MS/MS has become the preferred method for analyzing stanozolol and its metabolites in routine doping controls. Nevertheless, GC–MS/MS–based applications are still in place, and extracting urine samples with mixed C‐8‐ and strong cation exchange sorbents instead of commonly employed LLE was shown to improve detection limits to 0.1–0.25 ng/mL [65], especially when employing a capillary column featuring a 0.33‐μm film thickness (dimethyl polysiloxane capillary column, 25 m, 0.2‐mm inner diameter) and a Q/orbitrap mass analyzer. It was further suggested that a transient matrix effect, exhibited by the addition of high‐boiling protectants such as polyethylene glycol 400, could enhance signal abundances of AAS when analyzed by GC–MS/MS–based methods [66], but whether that translates from (highly concentrated) spiked blood samples also to trace analyses in urine is yet to be shown.

Identifying and characterizing metabolites, especially those of AAS, are a complex and challenging task, particularly when considering the highly nonpolar structures, variety of metabolically accessible sites within the steroidal scaffold, the stereochemical options, and different phase II conjugations. Streamlining workflows toward unknown metabolite detection in post‐administration urine samples was the subject of a study presented by Koomen et al., who employed LC‐ion mobility (IM)‐HRAM analysis in a proof‐of‐concept investigation with oxymetholone and methyl‐1‐testosterone [67]. Urine samples collected after a single oral dose of 150 mg of oxymetholone or 10 mg of methyl‐1‐testosterone were solid‐phase‐extracted and injected onto a C‐18 analytical column (2.1 × 75 mm, 1.7‐μm particle size) for chromatographic separation using water and methanol as solvents A and B, respectively, both containing 0.1% formic acid and 1 mM ammonium formate. The effluent was interfaced via negative ESI to a drift tube ion mobility‐Q/TOF instrument, and the generated data were processed in two protocols of either fully untargeted metabolomics or pseudo‐targeted metabolomics nature. Collision cross‐section (CCS) filtering (fractionating molecules by their CCS) combined with either mass defect analysis or temporal response profiling of observed features facilitated the identification of potentially relevant metabolites, upon which further MS‐based, nuclear magnetic resonance (NMR) spectroscopy–based, and/or chemical synthesis–based characterization steps can follow.

Synthetic reference materials are an important aspect of routine doping controls and, in consideration of the aforementioned metabolic variability, the production and evaluation of such products represent a discrete facet of anti‐doping research. In the context of steroidal reference compounds, Kuroe et al. reported on the successful preparation of a methanolic reference solution of 3β,4α‐dihydroxy‐5α‐androstan‐17‐one, outlining the applicability and utility of quantitative NMR spectroscopy and LC with ultraviolet detection in characterizing the product and its stability [68]. The unexpected availability of a 17α‐methyltestosterone metabolite referred to as 17,17‐dimethyl‐18‐nor‐5α‐androst‐13‐en‐3β‐ol was presented by Zhu et al., who incidentally obtained the metabolic product in a commercially available “supplement” labeled to contain the AAS desoxymethyltestosterone [69]. Characterized by NMR spectroscopy and GC‐EI‐MS/MS, the compound's composition was proven, providing essential information for result management processes, especially when the plausibility of an athlete's explanation of an AAF is assessed and the presumably ingested substance deviates substantially from the analytically determined metabolite(s). Also, the stability of AAS substances in solution has been investigated as reported by Martinez‐Brito et al., with the examples 6α‐chloro‐testosterone, 6β‐bromo‐androstenedione, and 6‐oxo‐androstenedione in methanol and dimethylsulfoxide [70]. Whereas 6‐oxo‐androstenedione did not exhibit signs of instability within 90 days at +4°C and −20°C, both 6α‐chloro‐testosterone and 6β‐bromo‐androstenedione showed considerable degradation (mostly to the dehalogenated analogs) at +4°C with a substantially more pronounced decomposition in dimethylsulfoxide, indicating that stock and working solutions (preferably in methanol) should be kept at −20°C.

### 3.2 Steroid Profiling and Detection in Urine and/or Blood

By means of the athlete biological passport (ABP) and its steroidal module, the use of pseudoendogenous compounds such as testosterone (T) can be detected. Given the considerable differences between athletes' urinary steroid profiles, the longitudinal monitoring of specific markers per individual has proven to be a sensible approach, and yet some markers (or ratios of markers) might be superior or inferior depending on the route of drug administration and/or the respective athlete's genetic predisposition. For instance, Coll et al. investigated the impact of a single oral dose of 80 mg of T undecanoate on the urinary steroid profile of 13 volunteers, 11 of which were defined as UGT2B17 *del/del* and two as UGT2B17 *ins/del* individuals [120, 121]. In a first study, it was observed that, among the commonly registered markers including T, epitestosterone (E), 5α‐androstane‐3α,17β‐diol (5αAdiol), 5β‐androstane‐3α,17β‐diol (5βAdiol), androsterone (A), and etiocholanolone (Etio), which were determined by means of established analytical protocols based on enzymatic hydrolysis, LLE, trimethylsilylation, and GC–MS/MS detection, the 5αAdiol/E ratio performed best in identifying steroid profile alterations [120]. Also, all samples that were flagged as atypical within the individual steroid profiles were also confirmed as AAF by GC/combustion/isotope ratio mass spectrometry (GC/C/IRMS). Yet, the detection windows were found to be limited to 24–48 h post‐administration, and alternative metabolites, especially sulfoconjugated urinary steroids, were assessed as to their potential to improve anti‐doping detection capabilities [121]. Sulfates of T, E, A, Etio, 5α‐dihydrotestosterone (DHT), epiandrosterone (epiA), dehydroandrosterone (DHA), 5α‐androstane‐3β,17β‐diol, 4‐androstene‐6β‐ol‐3,17‐dione, and 11‐oxo‐etiocholanolone were chosen as target analytes, determined by LC–QqQ–MS/MS analysis. Here, sample preparation consisted of weak cation‐exchange SPE, and extracts were chromatographed on a C‐18 analytical column (2.1 × 100 mm, 1.8‐μm particle size) with water and methanol (both containing 1 M ammonium formate/formic acid) as solvents A and B, respectively. The MS was operated using polarity switching ESI and MRM mode, allowing for limits of detection (LODs) between 0.4 and 8.3 ng/mL. These LODs enabled the sensitive monitoring of complementary markers and ratios thereof, and especially the ratios of 5α‐androstane‐3β,17β‐diol sulfate/DHA sulfate and epiA sulfate/DHA sulfate yielded superior detection windows by producing atypical steroid profile data points, thus flagging suspicious samples up to 144 h post‐administration.

Direct evidence for the exogenous origin of naturally occurring urinary steroids can be provided by the aforementioned IRMS, for which sample preparation and adequate chromatography preceding the IRMS are essential. Here numerous advances have been made as summarized in reviews and perspectives by Li et al. [122] as well as Polet et al. [123], who outline the importance and added value of IRMS data for anti‐doping testing as well as the progress in optimizing LC‐IRMS instruments. While the technology of LC‐IRMS is currently still inferior in terms of sensitivity when compared to GC/C/IRMS applications, it does not require analyte derivatization and can hence become an interesting complement to today's routinely employed methods.

Until then, targeting testosterone esters in doping control blood samples, more precisely in serum or DBS, has been proposed as an alternative approach. In an interlaboratory study, Langer et al. demonstrated the traceability of T propionate, phenylpropionate, decanoate, and undecanoate in DBS at 2 ng/mL, regardless of the employed sampling device, i.e., cellulose‐based or synthetic polymer‐based [124]. Moreover, the comparability with serum sample analyses was presented. The participating four laboratories did not harmonize their test methods; however, DBS extraction with organic solvents followed by concentration and derivatization (Girard T or P reagents) and subsequent LC–HRMS or LC–MS/MS was used in all applied analytical protocols. The effect of sampling device, extraction solvents, and derivatization reagents on recovery, matrix interference, and, eventually, detection limits of steroid esters in DBS was systematically investigated by Mazzarino et al. [125] Three different cellulose‐based DBS cards as well as two synthetic polymer‐based DBS devices were spiked with whole blood enriched with a total of 27 different boldenone, nandrolone, and testosterone esters before being subjected to extraction into various individual and combined organic solvents prior to derivatization of the target analytes with either methoxyamine, Girard T or P reagent. The chromatographic separation of the compounds of interest was conducted on a C‐18 analytical column (2.1 × 100 mm, 1.8‐μm particle size), using water and methanol (both containing 0.1% formic acid and 2 mM ammonium formate) as solvents A and B, respectively. The effluent was introduced into the QqQ‐based MS via positive ESI, and analytes were identified using MRM. This comprehensive dataset demonstrated that all variables, i.e., sampling devices, extraction solvents, and derivatization reagents, affect the respective LODs of individual target analytes, which is why an ITP based on methanolic extraction followed by Girard P or T derivatization was recommended. Confirmation procedures were, however, suggested to consider alternative combinations in order to provide best‐possible LODs for each steroid ester tested in this study. In order to assess the traceability of a transdermally applied T ester using DBS, an authentic human administration study with T propionate (in combination with methyltestosterone) and DBS sampling was presented by Miyamoto et al. [126] Five healthy male volunteers applied 1.5 mg of T propionate (together with 3 mg of methyltestosterone), formulated and commercialized as a hair growth product, on 5 consecutive days onto the skin below the chin. DBS and whole blood samples were collected up to 625 h after the last steroid application timepoint, and with an assay LOD of 0.1 ng/mL for both analytes, detection windows of 97 h were reported. The sample preparation consisted of two consecutive liquid extractions of cellulose‐contained DBS using a combination of *tert*‐butylmethyl ether/methanol/2‐propanol. The volumes were combined and dried, and the dry residue derivatized with methoxyamine before analysis by LC–QqQ–MS/MS. Here, a C‐8 analytical column (2.1 × 100 mm, 1.7‐μm particle size) and 0.1% formic acid (solvent A) and acetonitrile (solvent B) were employed to separate the analytes prior to introduction via positive ESI into the MS, which was operated in MRM mode.

In consideration of the growing challenges associated with different doping scenarios, especially concerning the misuse of pseudoendogenous steroidal preparations, the question of whether or not the exclusively targeted analytical approaches commonly used in doping control analyses are the best option for future anti‐doping efforts has remained unanswered. Complementary/alternative options are continuously being investigated, for example, by Salamin et al., who profiled the lipidome of 14 female study participants who underwent a 28‐day treatment with 10 mg of transdermally applied testosterone per day [71]. Pre‐, intra‐, and post‐administration, blood samples were collected and subjected to targeted lipidomics analyses. A total of 597 lipid species was considered in this study, few of which (predominantly esterified and ether‐linked lysophosphatidylcholines) showed significant changes attributable to the testosterone treatment. Since the observed changes were modest, an immediate benefit for anti‐doping purposes was not identified, but a first and previously unexplored dataset was produced.

### 3.3 Other Anabolic Agents

Other anabolic agents accounted for a total of 173 AAFs in 2023 [127], including 34 occurrences of clenbuterol and 139 findings of SARMs such as enobosarm (69 occurrences), LGD‐4033 (34 occurrences), RAD‐140 (18 occurrences), S‐23 (17 occurrences), and AC‐262,536 (1 occurrence), and concerns have been raised with regard to the accessibility [128] as well as missing information on the acute and long‐term toxicity of numerous SARMs. Fijalkowska and Jurowski therefore presented an in silico approach in support of predicting toxicological endpoints on the example of the SARM ACP‐105 [72, 73]. Using various algorithms (primarily based on structural information about the compound and predicted interactions with biological targets), a moderate probability of genotoxicity, skin and eye irritation, and cardiovascular risks has been identified. These have been found to be due to the inhibitory effect of the substance on the hERG potassium channel. However, confirmation of the computed data by biological experiments and inclusion of potentially active metabolites of ACP‐105 was seen as essential additional aspects to corroborate and expand the assessment.

**FIGURE 1:**
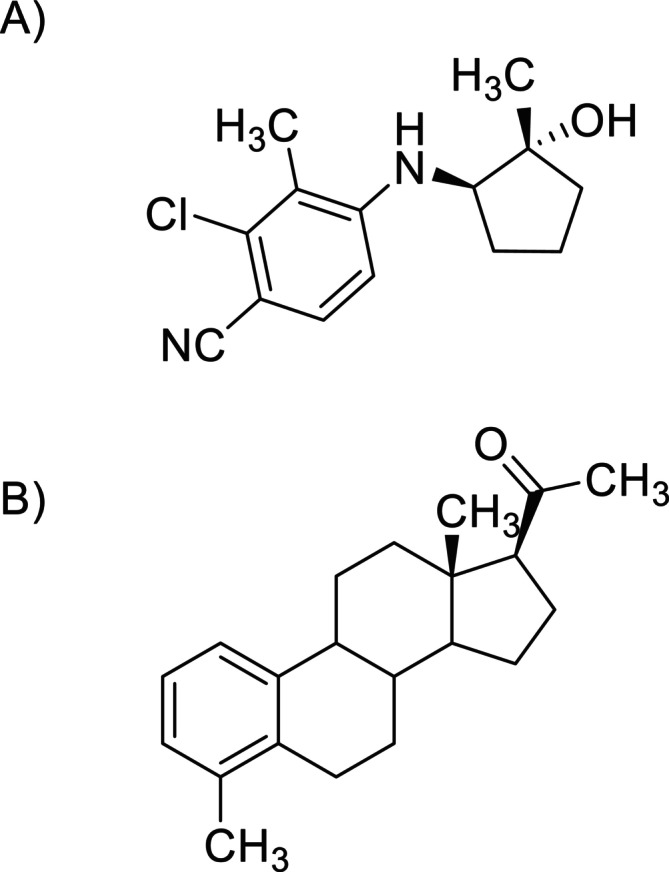
Chemical structures of the SARMs (A) LY305 and (B) S42.

In addition to the aforementioned SARMs, new (and yet less prominent) representatives of this class of prohibited substances were the subject of metabolism studies. For example, Kobidze et al. reported on the synthesis and in vitro metabolic conversion of LY305 (Figure 1A), an investigational SARM, currently under development for potential future transdermal application [74]. Exposed to human liver microsomal preparations, a total of 18 phase I and II metabolites was detected, largely resulting from hydroxylation, dehydrogenation, oxygenation, and/or glucuronidation, as well as combinations of these metabolic reactions, albeit predicting which biotransformation product might be the most representative for an implementation into routine doping controls is difficult, including the monohydroxylated and glucuronidated species of LY305 into existing testing approaches for SARMs is recommended, also in consideration of the compound's structural similarity to SARMs such as LGD‐4033 and SARM 2f.

The SARM S42 is, in contrast to LY305, of steroidal nature (Figure 1B), and assigning structures to its in vitro–derived phase I metabolites therefore poses a complex challenge as presented by Wen et al. [75] In a comprehensive study, both native S42 and a 7‐fold A‐ and B‐ring deuterated analog were subjected to human liver microsomal preparations, and formed metabolites were studied using GC‐orbitrap MS/MS experiments. In order to furnish mass spectral interpretation, three potential metabolites were chemically synthesized including 20‐OH‐S42, 6β‐OH‐S42, and 7α‐OH‐S42, and in combination with distinct fragmentation patterns of the trimethylsilylated analytes, eight metabolites were identified. Here, especially A‐ring mono‐hydroxylated species but also other *bis*‐ and *tris*‐hydroxylated metabolites were observed, for which now sufficient information exists to include dedicated precursor/product ion pairs into routine doping control analytical methods.

Notwithstanding the need to further improve and expand anti‐doping testing procedures in general and for new/emerging compounds, producing data on elimination profiles of both established and novel drug candidates is of particular relevance, especially for result interpretation and management. In that context, Alhalabi et al. investigated the elimination of the SARM S‐23 after oral ingestion, mimicking the scenario of athletes using an S‐23–contaminated dietary supplement [129]. Urine samples were collected from five male study participants per intervention, which represented single and repeated intake of 1, 10, or 50 μg of S‐23. Here, a total of 18 phase I and II metabolites were monitored using an LC–ESI‐Q/orbitrap system. Chromatographic separation was accomplished on a C‐18 analytical column (4.6 × 50 mm, 2.7‐μm particle size) with 5 mM aqueous ammonium acetate (containing 0.1% formic acid) and acetonitrile as ssolvents A and B, respectively. The MS was operated using negative ESI and full scan as well as PRM, allowing for an LOD of 1 pg/mL for S‐23. When urine samples were subjected to enzymatic hydrolysis and SPE prior to analysis, S‐23 was traceable up to 253 h (on average) when volunteers ingested a single dose of 1 μg of the SARM, and 50 μg of S‐23 administered on 5 consecutive days resulted in detection windows of 544 h (on average), illustrating the considerable analytical retrospectivity for S‐23, and thus, the low amounts that can result into a reporting of an AAF in sports drug testing. The relevance of transdermal exposure to SARMs rather than the oral intake was investigated by Korsmeier et al. [130] Here, study volunteers were applied once with 10 or 50 μg of S‐23, RAD‐140, and LGD‐4033 (introduced into 200 and 300 mg, respectively, of a dermal cream containing dimethylsulfoxide as penetration enhancer), and urine samples were collected up to 75 days post‐application. Urine sample preparation as well as analytical conditions were identical to those employed by Alhalabi et al. [129] (vide supra), with the addition of LGD‐4033 and RAD‐140 for which LODs at 28 and 5 pg/mL were validated. Under the chosen scenarios, AAFs for S‐23 and LGD‐4033 would have been concluded for up to 16 and 12 days (10‐μg dose), respectively, 25 and 24 days (50‐μg dose), whereas RAD‐140 was traceable primarily after exposure to 50 μg for up to 9 days, further corroborating that administration (or exposure) routes other than oral administrations should be considered when following‐up on AAFs in doping controls.

Flanking follow‐up investigations with analyses of alternative matrices such as hair has been reported at various occasions in the past, and also here knowledge on elimination or deposition, stability, and traceability is critical for result interpretation. With regard to ostarine (enobosarm/S‐22), Alvarez and Etting described the detection of the analyte in an athlete's hair sample 6 months after the ingestion of 20 mg of the SARM [76], providing first insights into the amounts that are required to allow for detecting ostarine ingestion by means of hair analysis with a given method limit of quantification of 0.05 pg/mL. Further details and, where possible, controlled studies are desirable to furnish the knowledge and thereby utility of complementary analyses in sports drug testing.

## 4 Peptide Hormones, Growth Factors, Related Substances, and Mimetics

### 4.1 Erythropoietin‐Receptor Agonists and Hypoxia‐Inducible Factor Activating Agents

Erythropoietin (EPO)‐receptor agonists (ERAs) have continued to contribute to WADA's AAF statistics, with in‐competition tests accounting for more than 60% of the findings as recently reviewed and summarized by Equey et al. [131] With performance‐enhancing effects of ERAs on athletes, especially in endurance sport categories, being comprehensively documented [132], and controlled studies on adverse effects of ERA use, e.g., on the cardiac morphometry underlining the importance of prohibiting ERAs in elite sport [133], also the optimization of anti‐doping testing approaches has been pursued in various regards. Regardless of whether urine or blood (i.e., serum, plasma, DBS) is tested for ERAs such as recombinant human EPO (rhEPO) and its analogs, the test matrices are commonly subjected to affinity‐based purification strategies that support the concentration and purification of the rather heterogeneous [134] target analytes from the doping control sample.

Here, new options complementing existing materials were presented, for instance, by Schwenke et al., who reported on the performance of the MAIIA anti‐EPO antibody 7D3 concerning its application to human urine [77]. Average recoveries of rhEPO, NESP, CERA, and EPO‐Fc from urine were determined at ca. 49%, 44%, 67%, and 22%, respectively, and LODs were found at ca. 10.7%, 12.0%, 10.8%, and 10.2%, respectively, of the applicable minimum required performance level (MRPL) [135], demonstrating the utility of this capture antibody for routine doping control purposes. The fact that also a single microdose of rhEPO administered at 15 IU/kg body weight is traceable both in urine and DBS by means of ITP and CP employing the antibodies 3F6 and 7D3, respectively, was corroborated by Heiland et al. [78] Five male volunteers participated in this study, and four returned presumptive AAFs up to 72 h post‐administration, with all samples (except for one) being confirmed using the CP approach. Concomitantly collected DBS returned PAAFs up to 48 h post‐administration, and lower detection rates were primarily attributed to the low sample volumes provided by two spots of 17 μL each.

Reichel et al. pursued an alternative approach employing magnetic nanoparticles noncovalently equipped with a polyclonal anti‐EPO antibody for isolating ERAs from urine or blood [79]. From a total of more than 50 monoclonal and polyclonal candidate capture antibodies, the polyclonal rabbit anti‐mouse EPO antibody (#51099‐T42) yielded best test results, including recoveries of rhEPO, NESP, CERA, and EPO‐Fc between ca. 63% and 104% and LODs below 10% of the applicable MRPL. In addition, the presented method was reported as considerably more cost‐effective than commercialized immunoaffinity purification kits for ERAs, an aspect that has received growing attention also in the context of routine sports drug testing programs. Employing a different antibody (rabbit anti‐human EPO, LS‐C11323) but a similar strategy, Miller et al. outlined the particular robustness of capillary serum samples for rhEPO analyses [80]. Microvolumetric specimens were collected both from athletes as part of their routine doping control program as well as a controlled administration study (15 IU/kg body weight), and samples were subjected to various storage conditions to assess the analytes' stability in serum (as opposed to urine). These conditions included storage at ambient temperature for up to 29 days, cold storage followed by freezing, etc., and aliquots of 100 μL were analyzed using routine protocols for ERA detection. Of note, all athletes' samples showed the presence of endogenous EPO (i.e., no signs of degradation), and except for those samples that were exposed to conditions of 50°C, reliable analytical data were obtained also for all other specimens.

Another potential affinity‐based purification strategy was described by Citartan et al., who reported on the production of RNA‐derived aptamers recognizing EPO as well as CERA [81]. Utilizing the in vitro approach referred to as Systematic Evolution of Ligands by Exponential Enrichment, a 71‐mer and its truncated 58‐mer analog aptamer were produced, which were immobilized to magnetic nanoparticles to serve as capture elements for EPO from biological matrices. The observed dissociation constants suggest utility for method development in routine doping controls, but essential parameters such as specificity, recovery, and LODs when employed in anti‐doping analytical assays require further assessment.

Eliminating EPO originating from the human *EPO c.577*del variant from urine for doping control purposes was the subject of an investigation by He et al. [82] As a rare but yet naturally occurring EPO variant, it might interfere with the result interpretation of rhEPO in human urine, which is why approaches facilitating its depletion from doping control samples have been desirable. Therefore, a peptide composed of a sequence differing between wild‐type human EPO and its variant (VAR‐EPO) was synthesized and used for immunization of New Zealand rabbits, and candidate antibodies were selected for expression and production, yielding a suitable clone referred to as TYJ9‐R0016 that was implemented into a combined reverse‐normal immunopurification protocol. Applied to VAR‐EPO–spiked samples as well as controlled elimination studies, it was shown that two cycles of reverse‐immunopurification allowed for eliminating VAR‐EPO almost entirely from urine, but not from serum, and the authors recommended following up on presumptive AAFs for rhEPO using urine rather than blood samples. In addition, to implement means to assess the successful immunoaffinity purification of the reverse‐normal purification process, Niu et al. reported on the use of a branched polyethylene glycol derivative conjugated to VAR‐EPO, which is recognized by the TYJ9‐R0016 antibody [83]. Spiked into doping control samples prior to extraction, its detection corroborates the functionality of all preparation and imaging steps of the overall procedure.

Whether or not oral fluid introduced into a urine sample, as a means of sample manipulation, has an effect on the analysis of ERAs was investigated by Garzinsky et al. [84] In a comprehensive study, numerous variable parameters (e.g., oral fluid volume, preprandial/postprandial oral fluid, incubation duration, etc.) were assessed as to their impact on the stability of ERAs in urine, and in approximately 31% of all analyzed specimens, the introduction of oral fluid into the urine sample caused interferences that did not allow adequate result interpretation. The presence of oral fluid in urine was, however, proven by the analysis of peptides originating from salivary proline‐rich proteins (saPRPs), which would allow for uncovering any manipulation attempts with oral fluid. As little as 20–200 μL of oral fluid in 100 mL of urine was shown to be traceable by targeting specific peptides of saPRPs with LC–HRMS/MS approaches. Here, a C‐18 analytical column (3 × 50 mm, 2.6‐μm particle size) operated with water (solvent A) and acetonitrile (solvent B), both containing 0.1% formic acid and 1% dimethylsulfoxide, was used to separate the analytes of interest. Following ESI in positive mode, the peptides were identified using a Q/orbitrap mass analyzer in full scan and PRM mode, providing the required information to confirm or to rule out the presence of saPRPs in human urine.

Although most studies aimed at improving direct detection methods for rhEPO and its analogs, also indirect approaches utilizing biomarker‐based tests were further pursued. Salamin et al. investigated the effect of single (CERA, 200 μg sc) and multiple (Dynepo, iv or sc, 3 × 5000 IU followed by 3 × 2500 IU) administrations of ERAs on the fraction of polar species of the human metabolome, targeting a total of 196 preselected analytes [85]. Here, serum and plasma samples (collected up to 27 days) were subjected to protein precipitation, and supernatants were injected onto a hydrophilic interaction amide column (2.1 × 100 mm, 1.7‐μm particle size). solvents used were 20 mM ammonium formate (A) and acetonitrile (B), both containing 0.1% formic acid, and the effluent was directed via positive ESI to a QqQ‐based MS system operated in scheduled MRM mode. Various metabolites were found to be significantly altered in abundance, with inosine and hypoxanthine being most prominently affected, indicating that further studies as to their utility as potential biomarkers are warranted.

**FIGURE 2:**
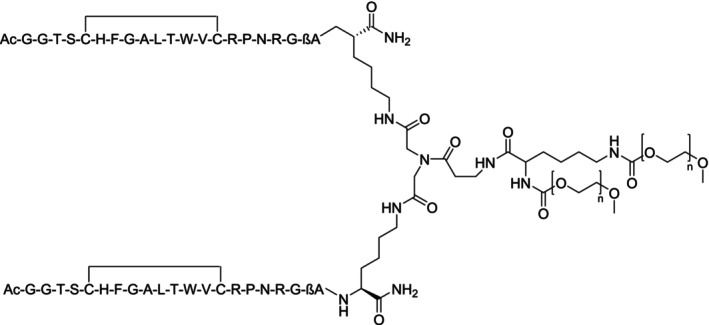
Composition of the EPO‐mimetic drug pegmolesatide.

Whether or not indirect and biomarker‐based approaches are suitable also for the detection of the misuse of novel EPO‐mimetic agents such as pegmolesatide (Figure 2) is yet unclear, but a direct analytical assay was presented by Liu et al. [86] The drug, approved in 2023, is composed of a homodimeric peptidic core fused to polyethylene glycol, and the authors employed magnetic nanoparticles coated with the recombinantly produced EPO receptor to isolate the EPO mimetic from human serum. Upon extraction, the analyte was digested by trypsin, and proteotypical peptides were used as targets for subsequent detection on a nanoLC‐Q/orbitrap MS. The analytical column contained a C‐18 stationary material (75 μm × 150 mm, 2‐μm particle size) and 0.1% formic acid (solvent A) and 80% acetonitrile/20% solvent A (yielding solvent B) were used as eluents. The MS was operated in positive nanoESI mode with targeted MS/MS experiments, allowing for an assay LOD of 2 ng/mL. In consideration of pphase II clinical trial data suggesting serum concentrations of ca. 50 ng/mL and drug half‐lives of ca. 50–70 h, the method is fit‐for‐purpose and use in routine doping controls.

In consideration of the potential (endurance) performance‐enhancing effects of hypoxia‐inducible factor (HIF)–activating agents, in particular, substances with prolyl hydroxylase‐inhibitory effects have been prohibited by WADA since 2011 [32], and recently, evidence for the underlying mechanism of performance enhancement exerted by the HIF activator roxadustat was provided by means of a mouse model [136]. Among the HIF‐activating agents, also cobalt and xenon are listed, and Postnikov et al. conducted administration studies to investigate the impact of either compound on the expression profiles of microRNAs involved in the human organism's regulation of the HIF‐1α transcription factor [137]. Healthy male volunteers received either 800 μg cobalt aspartate (equivalent to ca. 240 μg of cobalt) for 20 days once per day or inhaled a xenon/oxygen mixture (27:75, v/v) for 30 min per day on 5 consecutive days, and blood samples were collected prior to and 1 day after completion of the drug intervention program. Employing reverse transcription/quantitative PCR, 84 microRNA targets were analyzed, revealing hsa‐miR‐15b‐5p for cobalt and hsa‐miR‐378a‐3p as well as hsa‐miR‐491‐5p for xenon as significantly altered under the chosen conditions. It remains to be clarified to which extent these markers can contribute to anti‐doping routine analyses, e.g., also with regard to other HIF activators such as roxadustat, but at least their robustness against physiological hypoxia as a confounding factor was previously described. As an alternative and complementary option to determine the use of/exposure to roxadustat and other related HIF‐activating agents, keratinized matrices such as hair have been shown to retain such compounds, and also here further data, especially concerning factors influencing the incorporation, are desirable for a more comprehensive use in follow‐up investigations [138].

### 4.2 Testosterone‐Stimulating Peptides (In Males) and Growth Hormone, Its Analogs, and Releasing Factors

The effect of testosterone‐stimulating peptides such as triptorelin on various parameters determined in anti‐doping is well established, including, e.g., their influence on the urinary steroid profile. In light of their comparably low metabolic stability, information on biotransformation products enabling prolonged detection windows after drug use for urine and serum analysis are highly desirable in routine doping controls. Saardpun et al. reported on the detection of a triptorelin degradation product referred to as triptorelin [5, 6, 7, 8, 9, 10] in samples collected from prostate cancer patients who received a single intramuscular dose of therapeutic triptorelin pamoate of 11.25 mg [139]. Following a straightforward weak cation‐exchange SPE of urine, respectively, protein precipitation and mixed‐mode anion exchange SPE of serum, extracts were subjected to LC–HRMS/MS analysis. Different analytical instrumentations proved useful, with C‐18 analytical columns of either 2.1 × 100 mm (2.5‐μm particle size) or 2.1 × 150 mm (2.7‐μm particle size), both operated with 0.1% formic acid (containing 1% dimethylsulfoxide, solvent A) and acetonitrile (ssolvent B), and either ion trap/TOF or Q/orbitrap MS detection. The hexapeptide triptorelin [5, 6, 7, 8, 9, 10] was traceable in the studied patients up to 1 month after drug administration, indicating a longer analytical retrospectivity provided by this metabolite than the intact triptorelin, but whether this therapeutic drug regimen reflects also doping scenarios remains to be clarified.

Analyzing growth hormone (GH) is fundamental in routine doping controls but also in the context of public health considerations, given the still substantial number of confiscated illicitly sold GH products [140]. Besides recombinant human GH, the variety of available GH analogs, most of which feature prolonged durations of action, is continuously growing, and recent publications have outlined the importance of test methods complementing the commonly applied isoform differential immunoassay, which employs both permissive and highly specific monoclonal antibodies to allow for calculating ratios between the predominant GH isoform of 22 kDa and other variants.

For the long‐acting GH referred to as somatrogon, representing a fusion protein of the 22 kDa human GH and two copies of the C‐terminal peptide of human chorionic gonadotropin β‐subunit, an unexpectedly large fraction was detected in post‐administration urine samples, facilitating the development of urine analytical approaches for this specific long‐acting GH. Walpurgis et al. modified a previously established detection method for serum somatrogon by concentrating 2 mL of urine via ultrafiltration for subsequent affinity purification using GH receptor–coated magnetic nanoparticles [141]. Upon trypsin digestion, diagnostic glycosylated peptides were analyzed using a C‐18 analytical column (3.0 × 50 mm, 2.7‐μm particle size) operated with 0.1% formic acid (solvent A) and acetonitrile (solvent B), both containing 1% of dimethylsulfoxide. The LC effluent was electrosprayed into a Q/orbitrap‐based MS, operated in full scan as well as targeted single ion monitoring (tSIM) mode, allowing to detect somatrogon at 1 ng/mL in human urine. With this LOD, post‐administration urine samples collected from study volunteers who received a single dose of sc injected somatrogon at 0.66 mg/kg, the drug was traceable for 96 h. Of note, the isoform differential immunoassay commonly used for serum analyses in routine doping controls was found to be applicable also to urine samples, and although the concentration ratios produced by two so‐called REC kits for 66 reference urine samples was found between 0.8 and 2.4, somatrogon post‐administration urine samples returned REC2/REC1 concentration ratios of 111–1695, indicating a potential additional use of the methodology for sports drug testing purposes.

GH‐releasing factors such as GH‐releasing peptides (GHRPs) stimulate the secretion of naturally produced GH, which is why these lower molecular mass peptidic compounds are targeted with dedicated LC–MS(/MS) approaches in routine doping controls. Here, information on the metabolism of those GHRPs is important to ensure best‐possible analytical retrospectivity and specificity, and in that context, Pobee et al. investigated the in vitro biotransformation processes of alexamorelin [142]. Incubated with pooled human hepatocytes, mixtures of alexamorelin and putative metabolites were analyzed by LC–HRMS(/MS) for potential biotransformation products predicted using in silico computation. Separated on a biphenyl reverse‐phase analytical column (2.1 × 150 mm, 2.6‐μm particle size) using 0.1% formic acid and acetonitrile (containing 0.1% formic acid) as solvents A and B, respectively, peptidic analytes were identified using positive and negative ESI with Q/orbitrap MS detection. From a total of 21 hypothesized metabolites, only one C‐terminally truncated species (exhibiting the same composition as the GHRP hexarelin) was experimentally determined.

Independent of the means of artificial GH stimulation, biomarkers such as the IGF‐I and the *N*‐terminal propeptide of the Type III procollagen (P‐III‐NP) are expected to efficiently flag GH‐related doping scenarios. In order to eliminate interindividual variability, the endocrine module of the ABP employing IGF‐I and P‐III‐NP as decisive markers has been launched, and first results of a proof‐of‐concept study including 54 athletes (26 males and 24 females) from 16 sport disciplines were reported by de Figueiredo et al. [87] With harmonized analytical methods, here LC–MS/MS‐based quantification of IGF‐I and immunoassay‐based determination of P‐III‐NP, individual GH2000 scores proved stable over time; however, the announced discontinuation of the immunoassay outlined the relevance of suitable alternatives to the analytical platform or to the marker P‐III‐NP as such to ensure an informative longitudinal monitoring. Here, Huynh et al. reported on a new strategy allowing for the quantification of P‐III‐NP and other fragments of the Type III procollagen in human serum, using LC–MS/MS methods [88]. Here, 100 μL of serum was diluted before serum proteins were denatured, alkylated, and eventually hydrolyzed with trypsin, before stably labeled isotopologs of four target peptides of P‐III‐NP and P‐III‐CP were added. The peptide surrogates of P‐III‐P were extracted using monoclonal antibodies immobilized to magnetic nanoparticles, and the purified material was chromatographed on a C‐18 analytical column (2.1 × 50 mm) using 0.1% formic acid (containing 2% dimethylsulfoxide) and methanol (containing 2% dimethylsulfoxide) as solvents A and B, respectively. Diagnostic precursor–product ion pairs were monitored after positive ESI on a QqQ‐based MS in MRM mode, enabling the quantification of P‐III‐NP and P‐III‐CP in the ranges of 1.35–13.3 nM and 0.26–5.1 nM, respectively, and thus presenting an alternative approach to purely immunological analyses of the GH biomarker.

## 5 β_2_ Agonists, Hormone and Metabolic Modulators, Diuretics, and Stimulants

Although the detection of most representatives of these classes of prohibited substances at relevant concentrations in routine doping controls, in particular those of low molecular mass, has long been accomplished, research into effect, metabolism, and disposition (including hair as a potential complementary test matrix [95, 143, 144, 145]) of such drugs in the context of sport and anti‐doping analyses has been further pursued. In addition, especially for higher molecular mass metabolic modulators, new comprehensive analytical approaches were reported.

### 5.1 β_2_ Agonists

In their recent work, Persch et al. demonstrated that inhaled β_2_ agonists have a significant effect on the heart of healthy men and women in a 10‐min time trial. They administered either cumulative doses of 1200 μg of salbutamol, 36 μg of formoterol, a combination of both, or a corresponding placebo preparation to the test subjects before a bicycle ergometer stress test [89]. The results demonstrated improved left ventricular systolic fraction values, which were enhanced when combining both inhaled β_2_ agonists, and the significance was higher in the female study group.

Hostrup et al. showed that orally administered salbutamol (24 mg), being a racemic mixture of the respective *R*‐ and *S*‐enantiomers, exhibits significant enantioselective disposition both in plasma and muscle tissue, with the (pharmacologically inert) *S*‐salbutamol being retained longer than its active counterpart [90]. Considering salbutamol's status as a threshold substance [91], in doping controls this observation might necessitate confirmatory analyses that allow quantification of both enantiomers separately [92].

Although the numbers of findings concerning higenamine in routine doping controls are low with six AAFs in 2023 [127], concerns as to unintentional intake or, as reported by Sakamoto et al., by metabolic conversion from coclaurine originating from Kampo medicines, still exist [93]. Indeed, previously unreported natural (dietary/medicinal) sources of higenamine and coclaurine continue to be identified, and concentrations up to ca. 200 μg/g and 2000 μg/g were observed. It remains to be clarified, though, if such products could cause an AAF as, to date, only mice in vivo studies exist.

### 5.2 Hormone and Metabolic Modulators

Within this category, i.e., S4 of the WADA Prohibited List, diverse drug classes are combined, ranging from aromatase inhibitors and anti‐estrogenic substance via agents preventing activin receptor IIB activation (so‐called IASPs: inhibitors of the activin receptor signaling pathway) to four subcategories of various metabolic modulators.

Ramatercept (or ACE‐031) represents a to‐date nonapproved recombinant fusion protein composed of a fragment of the human activin receptor IIB (ACVR2B) and the Fc‐part of the human IgG1. The composition allows ACE‐031 to act as a decoy activin receptor, which reduces the availability of ligands such as the negative regulators of muscle growth (e.g., myostatin) to the natural human activin receptor IIB, and numerous products claiming to contain ACE‐031 have been offered via online‐based providers, which were investigated as to their content by Reichel et al. [94] Fourteen products were acquired, 12 of which exhibited human activin receptor IIB immunoreactivity, which was proven to result from the presence of full‐length human activin receptor IIB rather than the claimed Fc‐fusion protein ACE‐031. Employing immunoaffinity purification with anti–ACVRIIB‐antibody–coated magnetic nanoparticles and subsequent SDS‐PAGE/Western blotting, a test method was developed to allow for analyzing serum and urine samples of male and female rats, who received a single subcutaneous dose (10 mg/kg body weight) of one of the 12 products sold as ACE‐031. The assay allowed for LODs of 1 and 2.5 ng/mL for serum and urine, respectively, and the administered product was traceable for up to 48 h using the developed approach. How these results translate to the detection of authentic ACE‐031 and human administration warrants further investigations.

Inhibition of the activin–receptor IIB activation can be accomplished also via other therapeutic strategies, including, e.g., antibodies directed against the ligands such as garetosmab, stamulumab, landogrozumab, and domagrozumab, or antibodies against the precursor of the active myostatin such as apitegromab. Here, multiplexed test methods were deemed relevant in order to address the challenge arising from this growing class of drug candidates, which Sakellariou et al. accommodated with a combined affinity‐purification and LC–HRMS/MS analytical approach [146]. First, magnetic nanoparticles were covalently coated with activin A, growth differential factor 11, myostatin, pro/latent myostatin, and a goat anti‐human ACVR2B antibody. Then, serum was protein‐precipitated with saturated ammonium sulfate, and the obtained pellet was recovered, diluted, and affinity‐extracted. The extract was subjected to trypsin digestion, followed by LC–HRMS/MS analysis. Here, separation of proteotypical peptides was accomplished on a C‐18 analytical column (3 × 50 mm, 27‐μm particle size) using 0.1% aqueous formic acid (solvent A, containing 1% dimethylsulfoxide) and acetonitrile (solvent B, containing 0.1% formic acid and 2% dimethylsulfoxide). The effluent was introduced into the MS via positive ESI, and diagnostic target analytes were monitored in tSIM and data‐dependent MS/MS mode, allowing for LODs for all substances between 10 and 50 ng/mL. Luspatercept (also a fusion protein‐based decoy receptor) post‐administration serum and urine samples were analyzed for proof‐of‐concept, and specimens collected from volunteers who received two subcutaneous injections of 0.25 mg/kg body weight within 3 weeks were found to contain the target analyte up to 70 days after the first injection, highlighting the flexibility of the analytical assay and the fitness‐for‐purpose concerning routine doping controls.

Focusing on three monoclonal antibodies, namely, landogrozumab, domagrozumab, and bimagrumab, Lee et al. reported on a protein G–facilitated affinity purification strategy followed by LC–field‐asymmetric ion mobility–MS/MS analysis of plasma and DBS [147]. Here, DBS samples (from 20 μL of blood) were extracted into a buffer solution (while 5 μL plasma aliquots were simply diluted) before purification with magnetic nanoparticles coated with protein G. The extract was trypsinized, desalted, and injected onto a nanoLC system equipped with a C‐18 analytical column (75 μm × 150 mm, 3‐μm particle size). Solvents used were 0.1% formic acid (eluent A) and 80% acetonitrile (containing 0.1% formic acid, solvent B), and the MS was operated in positive ESI mode with PRM. The accomplished LODs ranged between 100 and 1000 ng/mL for both matrices.

With regard to lower molecular mass drugs of the category S4, aromatase inhibitors such as letrozole have been mentioned as a predominant therapy or an alternative treatment among previous AAS users [148], both in therapeutic but also nonsupervised post‐cycle self‐treatment programs, with the latter largely utilizing drugs obtained from illicit sources. Here, presumed health risks originating from unregulated use and questionable product quality [149] outweigh potential concerns regarding comprehensive anti‐doping screening methods [150]; however, with respect to the detection of drug residues as a result of possible contamination scenarios, options of providing additional analytical data for the required case and result management were revisited. For instance, Postnikov et al. performed a pilot study with three volunteers consuming 900 mL of fresh, nonpasteurized milk obtained from emidonol‐treated cows, with emidonol consisting of meldonium and emoxypine [96]. Details of the dosing of the animal are limited, and the concentrations of meldonium and emoxypine in the consumed milk are unknown; nevertheless, the estimated concentrations and the ratio of both analytes determined in human urine could provide supporting information for doping controls, with emoxypine being present at considerably higher concentrations than meldonium at times that meldonium exceeded the applicable minimum reporting limit (MRL) of 100 ng/mL. Hence, monitoring emoxypine (glucuronide) was recommended, employing enzymatic hydrolysis and SPE of urine prior to injection onto a HILIC analytical column (3.0 × 100 mm, 1.7‐μm particle size). Solvents used were 10 mM ammonium acetate (containing 1% acetic acid, eluent A) and acetonitrile/methanol (3:1, v/v, eluent B), and following positive ESI, emoxypine was determined in SRM mode on a QqQ‐based MS. In addition, first data on the incorporation of meldonium into hair were presented by Kintz et al. [95], which warrant further investigation with controlled administration studies and, in the light of the emoxypine data presented by Postnikov et al., could be complemented by including the emidonol marker also into future hair analysis projects.

### 5.3 Diuretics and Stimulants

Most diuretics and stimulants are commonly monitored in sports drug testing urine samples as intact compounds, i.e., unmetabolized substances. In order to support the fact that a drug found in a doping control specimen was administered, phase I or II metabolites co‐detected in cases of AAFs could provide additional information for case management purposes. For chlorthalidone, Di Giorgi et al. conducted in silico and in vitro studies (with human hepatocytes) to produce analytical data potentially applicable to routine doping controls. From a total of 11 computed metabolites, two candidates were detected in hepatocyte incubates of the drug, which were tentatively attributed to a hydroxylated as well as a hydrogenated species of chlorthalidone, located at the phthalimidine moiety of the molecule [151].

In order to improve the estimation of urinary concentrations of target analytes, in particular those of stimulants, Wang et al. suggested an approach referred to as dual isotopic labeling [152]. Using 10 different stimulants including (among others) heptaminol and amfetamine, an approach was proposed that employs derivatization of the analytes with ^13^C‐labeled dansylchloride as internal standards, which are mixed to urine samples prepared with ^12^C‐composed dansylchloride. Therefore, routine doping control urine samples are centrifuged, filtered, and diluted prior to addition of the derivatizing agent. Upon completion of the derivatization, previously ^13^C‐labeled derivatives of the target analytes are added to the urine specimens, and aliquots are analyzed by LC–HRMS/MS. The compounds are separated on a C‐18 analytical column with the dimensions of 2.1 × 100 mm (1.8‐μm particle size) and 0.1% formic acid (solvent A, containing 10 mM ammonium formate) and acetonitrile (solvent B), before introduction via positive ESI into a Q/TOF‐MS–based instrument operated in MRM mode. LODs between 0.02 and 1 ng/mL were reported, and with the availability of stable isotope‐labeled internal standards in each analytical run, concentration estimations were found to be particularly robust and accurate. One reason for concentration estimations for stimulants in doping controls is the fact that stimulants are prohibited in‐competition only, and for result interpretation, MRLs apply with considerations of the estimated analyte concentrations. Whether or not these allow for differentiating out‐of‐competition use from in‐competition use was exemplarily studied by Nair et al. with amfetamine, methylphenidate, and modafinil [97]. Volunteers received single doses of the drugs at 20, 18, or 100 mg, respectively, once per day over 5 consecutive days, and urine, serum, DBS, and oral fluid were collected up to 48 h after the last dose. Urine samples were diluted and enriched with stable isotope‐labeled internal standards prior to LC–QqQ–MS analysis, whereas serum samples were first protein‐precipitated and concentrated before reconstitution and analysis. DBS were extracted into methanol with ultrasonication, prior to concentration and reconstitution, and oral fluid was diluted with methanol, centrifuged, concentrated, and reconstituted for subsequent analysis. Chromatography was done using a C‐18 analytical column (2.1 × 50 mm, 2.6‐μm particle size) with 0.1% formic acid (containing 10 mM ammonium formate, solvent A) and methanol (solvent B), and the MS was operated in positive MRM mode. Of note, all participants exceeded the urinary MRL of 50 ng/mL at 48 h post‐dosing, underlining the challenge of determining in‐competition use based on drug concentrations only; however, other matrices did not provide an immediate alternative either due to missing normalization in case of oral fluid and the absence of MRLs (or levels indicating the athlete being under the influence of the drug) for serum and/or DBS.

Differentiating the use of the stimulant meclofenoxate from exposure to nonprohibited chlorphenesin‐containing cosmetics or the muscle relaxant chlorphenesin carbamate was the subject of further investigations by several research groups. Lu et al. compared urinary metabolite profiles of 4‐chlorophenoxyacetic acid and 4‐chlorophenoxy‐2‐hydroxypropanoic acid observed after applications of either rinse‐off or leave‐on products (i.e., face wash, sunscreen or body lotion) as well as single oral dose of meclofenoxate [98]. The considerable concentration differences and metabolite profiles observed under the given settings supported the currently enforced proceedings and interpretation guidelines [99]. Likewise, the use of chlorphenesin carbamate was confirmed to produce a characteristic metabolite profile readily distinguishable from meclofenoxate use as presented by Xu et al. [100, 101].

## 6 Glucocorticoids and Manipulation of Blood and Blood Components

### 6.1 Glucocorticoids

In addition to those glucocorticoids mentioned under S9 of the WADA Prohibited List [32], vamorolone was discussed as relevant representative of this class of drugs, potentially warranting consideration in anti‐doping. Li et al. reported on metabolic products obtained by in vitro metabolism experiments using human liver microsomal preparations, which yielded a total of 10 phase I and two phase II biotransformation products, with structures tentatively assigned using LC–HRMS/MS data [102]. Most prominent metabolites were obtained by reduction of the C‐20‐located oxo‐function to α‐ and β‐oriented hydroxyl groups or dehydrogenation forming a C‐6‐C‐7 double bond, which were suggested to serve as target analytes for sports drug testing purposes. More data, in particular those from controlled administration studies, might be necessary to complement the preliminary findings, e.g., with regard to urinary metabolite profiles and applicable MRLs for this substance.

### 6.2 Manipulation of Blood and Blood Components

The hematological module of the ABP is a central tool of today's anti‐doping testing procedures to detect practices commonly subsumed as “blood doping.” Whole blood, collected via venipuncture, is analyzed in a strictly controlled and harmonized manner for a comprehensive set of parameters. In the light of the availability of novel microcapillary blood sampling devices, the option of combining data from conventional blood draws with those produced from capillary blood collections was assessed by Lewis et al., who compared results of venous blood samples and specimens collected with two different capillary sampling devices [153]. With the exception of platelet counts, excellent agreement was observed for 14 parameters and, consequently, also for the calculated OFF‐hr score, confirming the fitness‐for‐purpose of such sample collection devices for ABP purposes in routine doping controls.

Selected parameters of the ABP might be affected by scenarios and circumstances other than doping as debated, for instance, in the context of “simulated altitude” [154] or genetic predispositions [155], and although the status of carbon monoxide (CO) inhalation has been clarified meanwhile by WADA in the 2026 edition of the Prohibited List [156], CO's potential as doping agent has been the subject of controversial discussions [157, 158]. Another factor to consider might be glucocorticoids, which are prohibited in‐competition only; however, recent data suggest that repeated use of dexamethasone also affects parameters of the hematological module of the ABP. Requena‐Tutusaus et al. conducted a multidose oral administration study with 2 mg of dexamethasone twice per day over 5 days, and blood samples (collected up to 8 days after the first dose) were subjected to complete blood count analyses while applying standard ABP blood sample analytical conditions [103]. Also, the OFF‐hr score and abnormal blood profile score were calculated, as well as hemoglobin mass (Hb_mass_) and plasma volume were estimated using established predictive models. The obtained data showed a significant post‐treatment increase of the reticulocyte percentage (RET%) and immature reticulocyte fraction, indicating stimulated erythropoiesis. Also, abnormally low OFF scores were recorded, which could result in suspicious ABP findings in authentic doping control scenarios, and further studies appear necessary to follow‐up on these observations.

In addition to established parameters of the existing hematological module of the ABP, mRNA‐based biomarkers have been suggested as complementary measurands, in particular carbonic anhydrase 1 (*CA1*) and 5′‐aminolevulinate synthase 2 (*ALAS2*), in order to increase the passport's sensitivity, e.g., in detecting autologous blood microtransfusions. The quantification of these mRNAs (*CA1* and *ALAS2*) from DBS and their intraindividually stable levels in the absence of external factors such as doping scenarios was shown by Oliveira et al. [104] Based on that, Breenfeld Andersen et al. assessed the utility of these markers in a controlled autologous blood microtransfusion study with 47 trained individuals, 23 of which received 130 mL of packed red blood cells (RBCs) after donation of 450 mL of whole blood 4 weeks earlier [105]. Although the blood draw resulted in significant increases of *CA1*, *ALAS2*, and also RET% in all participants, only individualized longitudinal analyses of the mRNA markers allowed for outlier identification and, thus, indication of an autologous blood microtransfusion event. Hence, considering an implementation of (DBS‐based) *ALAS2* and *CA1* determinations into the ABP program was suggested. Of note, *ALAS2* appeared to be the superior marker when compared to *CA1*, whereas the latter has been seen particularly useful in detecting recombinant EPO administrations.

Although over 34,000 ABP blood samples were analyzed worldwide in 2023 for parameters of the hematological module [127], only 323 blood samples were subjected to analyses detecting homologous blood transfusion. Assuming that costs associated with standard analytical methods such as flow cytometry play a major role in that regard, Teixeira et al. evaluated whether conventional gel card kits commonly used in clinical practice would allow to detect double populations of RBCs featuring relevant minor antigens at relevant concentrations [106]. Depending on the donor blood percentage and the type of minor antigens expressed by the donor, the gel card test kits allowed for the detection of 3% (K, Jka, and Jkb) of homologous blood. If the recipient expressed C, c, E, and e but not the donor, then 5% of homologous blood was possible to visualize using the simple and cost‐effective approach. However, other surface antigens such as Fya, Fyb, S, and s did not provide sufficient sensitivity, and further studies with other antigens are planned in order to fully assess the potential of this alternative ITP option.

Besides manipulating the number of RBCs by transfusion events or ERAs, low molecular mass drugs enhancing the oxygen binding affinity of hemoglobin and, thus, artificially lowering the oxygen levels in the receiving tissue that can trigger erythropoiesis are also classified under category M1 of WADA's Prohibited List. One representative of such drugs is voxelotor, and Liang et al. investigated its in vitro and in vivo metabolism, identifying four target metabolites (in addition to voxelotor itself) that allow for detecting drug use for up to 20 days [159]. Five study participants received 10 mg of voxelotor orally, and urine samples were collected up to 28 days post‐administration. In consideration of data from combined stable isotope‐labeled and unlabeled voxelotor in vitro metabolism experiments, which facilitated the identification of a total of 32 biotransformation products, urine specimens were screened, exhibiting especially voxelotor, its reduced and glucuronidated species, as well as a degradation product resulting from the elimination of the 2‐hydroxybenzaldehyde moiety, as viable target analytes for routine doping controls. Also here, LC–HRMS/MS proved particularly suitable for the sensitive and specific detection of voxelotor and its main metabolites, with urine samples being merely solid‐phase extracted and injected into the LC–MS/MS system. Chromatographic separation was accomplished on a C‐18 analytical column (2.1 × 100 mm, 1.7‐μm particle size), using 5 mM ammonium formate with 0.05% formic acid as solvent A and methanol as solvent B. The MS was a Q/orbitrap system operated in positive ionization mode and full scan, respectively, PRM mode.

## 7 Chemical and Physical Manipulation

A major challenge to effectively applying sophisticated analytical methods to doping control samples is the substitution of specimens by other (not doped) individuals' urine. Such cases have been observed at various occasions in the past [160]. Likewise, confirming the authenticity of a doping control sample and ascertaining its origin from the intended athlete can be critical in situations where sample substitution or swap is suspected. The gold standard for corroborating donorship of a biological specimen is also in sports drug testing programs, based on DNA analysis, which has been frequently applied in follow‐up investigations. In that context, Akiyama et al. demonstrated that mitochondrial DNA (mtDNA) extracted from doping control urine samples allowed for testing and typing the hypervariable Regions 1 and 2 segmented into five fractions [107]. Due to the superior stability of mtDNA compared to nuclear DNA (nDNA), the presented approach is a useful complement to DNA typing methodologies utilizing autosomal short tandem repeats (STRs) of nDNA, especially when DNA degradation and corresponding allelic dropout events exist. However, with the inferior discrimination power of mtDNA, a two‐step protocol including STR analysis was proposed. Focusing on mtDNA analyses could further be useful in addressing the prohibited method of mitochondrial transplantation [161], for which animal models demonstrated significant increases in PGC‐1α mRNA expression and mitochondrial biogenesis [162], if mitochondrial heteroplasmy is detectable [163].

## 8 Gene and Cell Doping

**FIGURE 3:**
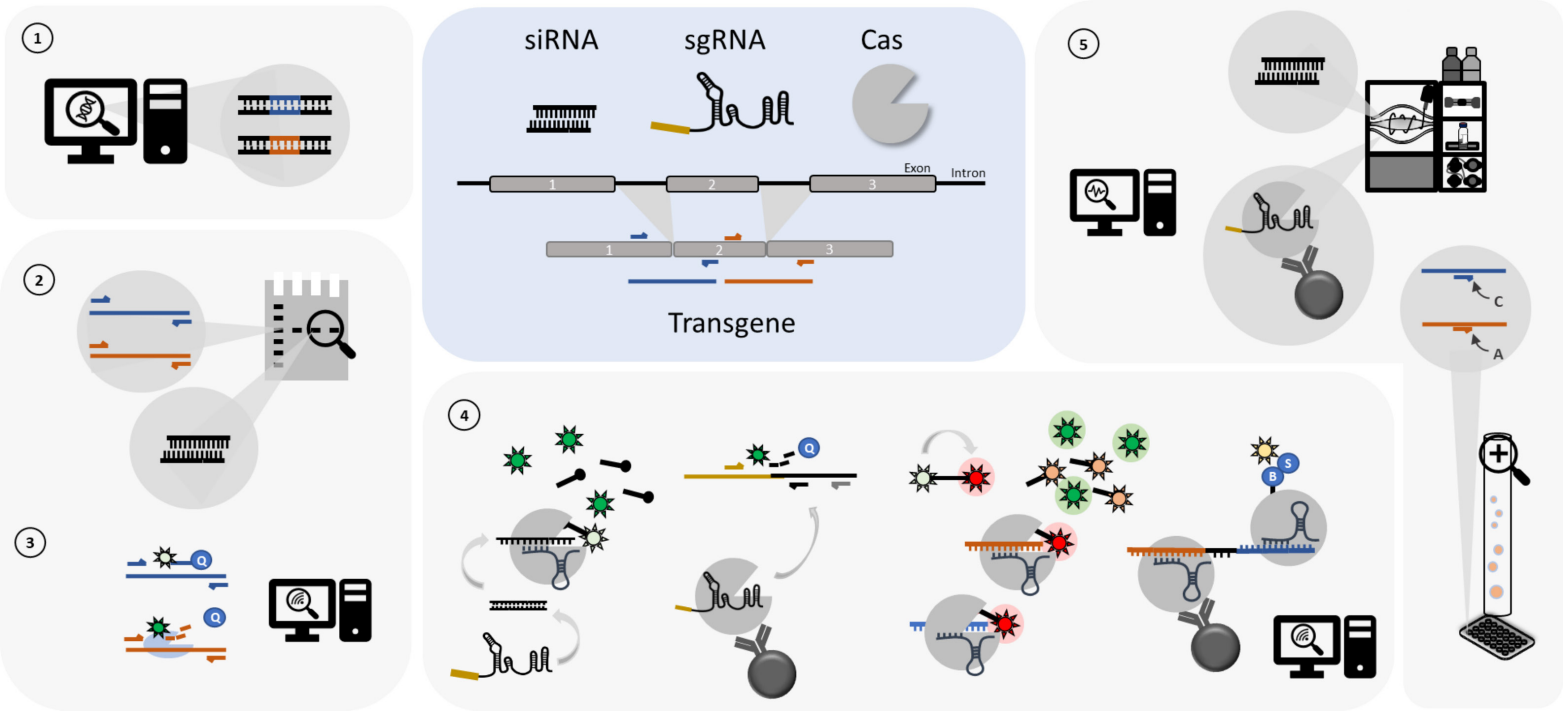
Simplified overview illustrating gene doping methods and corresponding detection strategies employing (1) next‐generation sequencing (NGS), (2) standard PCR combined with SDS PAGE, (3) qPCR/dPCR, (4) CRISPR/Cas systems, and (5) mass spectrometry (LC–ESI–HRMS, MALDI‐TOF MS).

Also, in 2024/2025, research aiming at establishing detection strategies for gene doping practices has been a priority topic in anti‐doping research. The rapidly evolving advances in modifying, manipulating, and influencing gene expression rates and the growing number of potential gene doping targets necessitate concerted efforts in order to minimize misuse of gene doping in sport by means of sensitive, specific, and comprehensive test methods. An overview summarizing gene doping strategies and corresponding detection options is given in Figure 3, illustrating common as well as distinct features of both gene doping methods and detection tools.

The therapeutic relevance of oligonucleotide‐based therapeutics in modern medicine was comprehensively reviewed by Parr and Keiler [108], underlining the enormous variety of approved drugs as well as novel drug candidates and potential gene targets necessitating anti‐doping consideration. Besides established products of antisense oligonucleotides, small interfering RNAs (siRNAs), microRNA mimics, and mRNA therapeutics, and small activating RNAs, as well as antibody–oligonucleotide conjugates, might require increased attention in the near future, and given the modified and nonnatural composition of the oligonucleotides, mass spectrometric approaches for their detection were deemed appropriate. Ren et al. further emphasized the importance of methods affecting chromatin conformations and thereby the upregulation or downregulation of specific gene expressions in the context of performance enhancement and the need for test method developments in that regard [164].

An analytical procedure that allows for detecting gene editing practices utilizing clustered regularly interspaced short palindromic repeats (CRISPR)–Cas9 with the target genes of myostatin (*MSTN*), α‐actinin 3 (*ACTN3*), *EPO*, and the EPO receptor (*EPOR*) was presented by Akiyama et al. [109] Employing immunoprecipitation of the CRISPR–ribonucleoprotein complex by means of magnetic nanoparticles coated with anti‐Cas9 antibodies, CRISPR RNA (crRNA) was isolated and purified from blood, followed by reverse transcription into cDNA and subsequent qPCR analysis using forward primers specifically recognizing the target gene–directed variable region of the guide RNA. Visualization of successful amplification and therefore presence of CRISPR–Cas9–based gene doping products were accomplished with a 6‐carboxyfluorescein (6‐FAM) fluorophore/black hole quencher 1 (BHQ1) probe, and amplicon confirmation was further achieved by electrophoretic analysis. Finally, proof‐of‐concept in vivo studies were conducted with intravenous and intramuscular injections of *MSTN*‐targeting CRISPR–Cas9 complexes as lipid nanoparticles into mice, and the CRISPR–Cas9 ribonucleoprotein was detectable for 12 and 24 h, respectively.

Yi et al. presented an approach that focused on the multiplexed detection of four transgenic target sequences including those encoding 22‐kDa GH, 20‐kDa GH, EPO, and IGF‐I by employing the aforementioned CRISPR–Cas–based approach as a sensitive and selective detection tool [110]. First, amplicons of exon–exon regions of the four model transgenes were generated by PCR directly in 5 μL of blood. After separation of cellular material, aliquots of the supernatant were incubated with CRISPR–Cas12a ribonucleoprotein complexes featuring target‐specific crRNA sequences, either in the presence of a 6‐FAM BHQ1 probe (indicating the presence of a transgene and functioning as ITP) or without a fluorescence probe for subsequent electrophoretic fragment pattern analysis as CP. Also here, a mouse model (repeatedly transfected via polyethyleneimine‐derived nanoinducer particles with human transgenic *EPO*) was used to provide proof‐of‐concept data, and with an estimated LOD of 2.5 copies in 5 μL of blood, the h*EPO* administration was traceable for up to 10 days.

Employing multiplexed exon–exon junction PCR amplification for seven transgenes with two or more assays per transgene followed by matrix‐assisted laser desorption ionization (MALDI)–TOF MS analysis, Naumann et al. presented a procedure allowing for the simultaneous detection of EPO, follistatin, GH, myostatin propeptide, IGFI, and the vascular endothelial growth factors A and D transgenes [111]. Prior to MALDI‐TOF MS measurements, amplicons were subjected to single‐base extension reactions, and the detection of single‐base extended primers confirmed the presence of a transgene. The applicability of the methodology to authentic post‐administration samples was demonstrated using plasma specimens obtained from a horse that received rAAV‐delivered human transgenic *EPO,* and with a validated LOD of 1500 copies per mL of blood, the transgene was confirmed until Day 11 post‐injection.

Aiming at providing a more comprehensive gene doping testing approach, Maehara et al. assessed the utility of largely automated DBS sample preparation (i.e., DNA extraction and library generation) and next‐generation sequencing (NGS) for sports drug testing purposes [112]. The whole genome analysis combined with appropriate bioinformatics enabled the identification of DNA fragments unequivocally attributable to transgenes as shown with a mouse model, where test animals received a retro‐orbital sinus AAV9_h*EPO* injection. Ten days post‐injection, blood samples were collected (showing significant increases in RBC count [10^4^/μL], hematocrit [%], and hemoglobin concentration [g/dL]) and analyzed with conventional TagMan qPCR as well as NGS assays. Both methods confirmed the presence of h*EPO*, and alignment analysis yielded a 100% match with the theoretical AAV vector sequence, demonstrating the applicability of this technology for gene doping analyses.

## 9 Conclusion

Updating, optimizing, expanding, streamlining, and refining anti‐doping analytical methods continue to be critical for modern sports drug testing programs, especially in consideration of the substantial pace at which therapeutic advances are made. Those, unfortunately, do not only ensure best‐possible therapeutic treatments for patients but also create opportunities for misuse by athletes aiming at gaining a competitive edge through illicit routes. Especially in the area of drugs/drug candidates and methods positively affecting muscle growth and/or endurance performance, vigilance as to new entities is of utmost importance. Furthermore, timely research into testing strategies and analytical procedures is vital to support global anti‐doping efforts in maintaining the integrity of sport.

**FIGURE 4:**
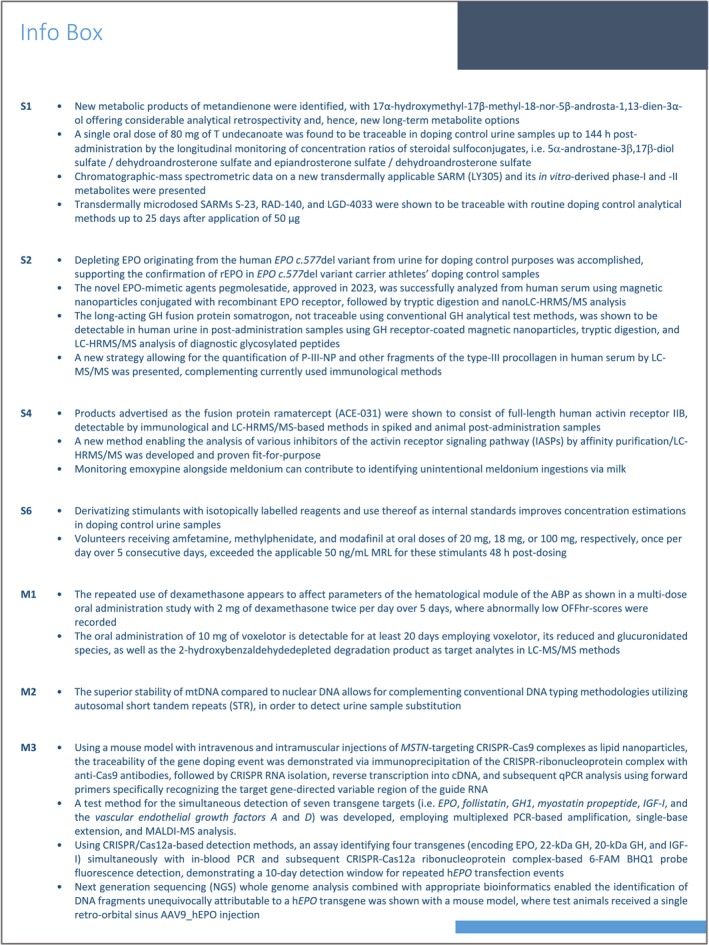
Info box on particularly relevant observations.

Over the 12‐month period covered in this 18th Annual Banned‐Substance Review*,* significant contributions to advancing analytical options beyond comprehensiveness and sensitivity, particularly in expanding anti‐doping sciences and applications toward new biotechnological options, were registered, and the observed key aspects of these are summarized in the Info Box in Figure 4.

## Funding

This work was supported by Manfred Donike Institut für Dopinganalytik, Federal Chancellery of the Federal Republic of Germany.

## Conflicts of Interest

The authors declare no conflicts of interest.

## Data Availability

Data sharing is not applicable to this article as no new data were created or analyzed in this study.
